# Supplementary material for: Subconcussive head impact exposure between drill intensities in U.S. high school football
Source: PLoS One. 2020 Aug 14;15(8):e0237800. doi: 10.1371/journal.pone.0237800 (PMC7428124; doi:10.1371/journal.pone.0237800)
Supplement: S1 Table — (DOCX) [file pone.0237800.s001.docx]

| **Supplemental Table 1: Bonferroni post-hoc results on 5 levels of contact** | | | | | |
| --- | --- | --- | --- | --- | --- |
|  | **Air** | **Bags** | **Control** | **Thud** | **Live** |
| **Cumulative impact frequency / player** |  |  |  |  |  |
| Air |  |  |  |  |  |
| Bags | -8.6  (-16.3, -1.0) p=0.019 |  |  |  |  |
| Control | -34.7  (-59.4, -10.1)  P=0.002 | -26.1  (-45.6, -6.7)  P=0.004 |  |  |  |
| Thud | -56.6  (-99.2, -13.9)  P=0.004 | -47.9  (-86.4, -9.5)  P=0.008 | -21.8  (-51.9, 8.2)  P=0.334 |  |  |
| Live | -101.1  (-155.4, -46.8)  P<0.001 | -92.5  (-143.2, -41.7)  P<0.001 | -66.4  (-108.7, -24.0)  P=0.001 | -44.5  (-88.4, -0.6)  P=0.045 |  |
| **Cumulative PLA / player, *g*** |  |  |  |  |  |
| Air |  |  |  |  |  |
| Bags | -194.1  (-383.2, -5.0) p=0.041 |  |  |  |  |
| Control | -787.2  (-1360.8, -213.6)  P=0.003 | -593.1  (-1064.2, -122.0)  P=0.007 |  |  |  |
| Thud | -1410.2  (-2522.5, -297.8)  P=0.007 | -1216.1  (-2218.6, -213.5)  P=0.010 | -623.0  (-1306.7, 60.8)  P=0.095 |  |  |
| Live | -2391.7  (-3713.4, -1070.0)  P<0.001 | -2197.6  (-3447.4, -947.9)  P<0.001 | -1604.5  (-2631.6, -577.4)  P=0.001 | -981.5  (-1943.2, -19.9)  P=0.043 |  |
| **Cumulative PRA / player, krad/s^2^** |  |  |  |  |  |
| Air |  |  |  |  |  |
| Bags | -19.1  (-37.3, -1.0)  P=0.034 |  |  |  |  |
| Control | -73.1  (-126.9, -19.3)  P=0.003 | -54.0  (-97.0, -10.9)  P=0.008 |  |  |  |
| Thud | -127.0  (-229.2, -24.9)  P=0.008 | -107.9  (-199.2, -16.7)  P=0.013 | -53.9  (-115.1, 7.3)  P=0.117 |  |  |
| Live | -212.4  (-336.4, -88.4)  P<0.001 | -193.3  (-309.0, -77.5)  P<0.001 | -139.3  (-230.2, -48.4)  P=0.001 | -85.3  (-166.0, -4.7)  P=0.033 |  |
| Note: Data are expressed as Mean difference (95% Confidence Interval for difference) and p-values. PLA, peak linear acceleration. PRA, peak rotational acceleration. | | | | | |
