# Supplementary material for: Subconcussive head impact exposure between drill intensities in U.S. high school football
Source: PLoS One. 2020 Aug 14;15(8):e0237800. doi: 10.1371/journal.pone.0237800 (PMC7428124; doi:10.1371/journal.pone.0237800)
Supplement: S2 Table — (DOCX) [file pone.0237800.s002.docx]

| **Supplemental Table 2: Cumulative head impact frequency and magnitude for the entire season** | | | | |
| --- | --- | --- | --- | --- |
|  | **Overall** | **Lineman** | **Hybrid** | **Skill** |
| Cumulative impact / player, count |  |  |  |  |
| Air | 3.50  (1.00-8.75) | 7.00  (1.00-9.00) | 4.00  (1.00-24.00) | 1.50  (0.75-10.75) |
| Bags | 11.50  (4.25-24.00) | 11.00  (5.00-32.00) | 15.00  (9.00-21.00) | 7.00  (2.75-27.25) |
| Control | 34.50  (13.25-69.50) | 48.00  (15.00-98.00) | 35.00  (14.00-49.00) | 14.00  (4.50-53.75) |
| Thud | 51.00  (23.25-73.50) | 51.00  (29.00-113.00) | 44.00  (22.00-69.00) | 32.50  (6.75-85.00) |
| Live | 100.50  (44.75-154.00) | 141.00  (93.00-304.00) | 131.00  (83.00-322.00) | 103.50  (24.00-179.75) |
| Cumulative PLA / player, *g* |  |  |  |  |
| Air | 76.62  (15.42-252.48) | 163.63  (16.38-259.80) | 80.27  (15.10-409.77) | 36.74  (10.34-239.54) |
| Bags | 264.07  (110.29-504.75) | 249.60  (109.53-543.40) | 359.91  (210.32-388.82) | 124.79  (51.99-629.52) |
| Control | 714.18  (313.43-1549.92) | 928.46  (345.34-2304.25) | 752.16  (334.17-1272.77) | 258.63  (79.61-1375.53) |
| Thud | 993.14  (464.35-2466.71) | 959.76  (690.73-3937.76) | 1026.52  (449.07-1435.04) | 731.81  (155.94-2170.89) |
| Live | 2165.14  (1029.32-3813.90) | 2188.60  (1749.63-5389.14) | 2403.35  (1538.30-6495.44) | 1192.06  (438.25-2947.68) |
| Cumulative PRA / player, krad/s^2^ |  |  |  |  |
| Air | 7.50  (1.48-24.01) | 15.21  (2.07-24.43) | 8.34  (1.29-44.87) | 3.61  (0.57-23.28) |
| Bags | 23.75  (9.20-50.64) | 23.66  (7.31-55.32) | 34.00  (16.46-36.59) | 16.41  (5.29-65.24) |
| Control | 63.90  (22.48-154.76) | 81.23  (23.33-231.48) | 64.68  (28.16-99.80) | 30.23  (8.67-138.07) |
| Thud | 92.63  (32.35-227.86) | 79.06  (45.41-350.56) | 96.52  (32.31-144.02) | 83.89  (18.99-200.43) |
| Live | 185.55  (83.93-382.00) | 179.08  (122.39-482.43) | 220.99  (99.44-562.53) | 105.42  (55.57-294.83) |
| Note: Data are expressed as Median (IQR). PLA, peak linear acceleration. PRA, peak rotational acceleration. | | | | |
