# Supplementary material for: Subconcussive head impact exposure between drill intensities in U.S. high school football
Source: PLoS One. 2020 Aug 14;15(8):e0237800. doi: 10.1371/journal.pone.0237800 (PMC7428124; doi:10.1371/journal.pone.0237800)
Supplement: S3 Table — (DOCX) [file pone.0237800.s003.docx]

| **Supplemental Table 3: Average peak linear acceleration and peak rotation acceleration** | | | | | |
| --- | --- | --- | --- | --- | --- |
|  | **Overall** | **Lineman** | **Hybrid** | **Skill** |  |
| Average PLA / impact, *g* |  |  |  |  |  |
| Air | 20.53  (14.46-28.46) | 23.38  (14.24-28.95) | 20.07  (15.10-24.32) | 20.02  (10.33-27.85) |  |
| Bags | 22.74  (17.66-23.95) | 22.12  (16.98-23.36) | 23.37  (22.60-25.92) | 20.41  (17.13-24.09) |  |
| Control | 22.72  (19.29-23.91) | 23.02  (19.34-23.67) | 22.93  (19.59-24.58) | 18.82  (15.93-24.81) |  |
| Thud | 22.84  (18.93-23.73) | 22.94  (18.82-23.41) | 20.89  (20.41-25.50) | 24.32  (16.36-25.76) |  |
| Live | 21.61  (19.82-25.09) | 20.95  (19.95-22.12) | 24.09  (19.78-28.63) | 24.77  (17.22-25.27) |  |
| Average PRA / impact, krad/s^2^ |  |  |  |  |  |
| Air | 2.03  (1.12-2.52) | 2.19  (1.06-2.53) | 1.67  (1.29-2.33) | 1.85  (0.57-3.05) |  |
| Bags | 1.92  (1.73-2.46) | 1.84  (1.46-1.97) | 2.12  (1.74-2.48) | 2.36  (2.13-2.55) |  |
| Control | 1.98  (1.65-2.22) | 1.75  (1.48-2.22) | 1.95  (1.80-2.04) | 2.16  (1.76-2.56) |  |
| Thud | 2.09  (1.49-2.30) | 1.84  (1.19-2.14) | 2.09  (1.47-2.34) | 2.47  (1.58-2.92) |  |
| Live | 1.98  (1.63-2.41) | 1.69  (1.30-2.04) | 2.27  (1.68-2.61) | 2.35  (1.68-2.98) |  |
| Note: Data are expressed as Median (IQR). PLA, peak linear acceleration. PRA, peak rotational acceleration. | | | | | |
