# Supplementary material for: Subconcussive head impact exposure between drill intensities in U.S. high school football
Source: PLoS One. 2020 Aug 14;15(8):e0237800. doi: 10.1371/journal.pone.0237800 (PMC7428124; doi:10.1371/journal.pone.0237800)
Supplement: Attachment — Submitted filename: Response to Reviewers.docx [file pone.0237800.s005.docx]

**Response to Reviewers**

We thank the reviewers and editor for their appreciation of the potential clinical significance of the current manuscript and their detailed and constructive comments, which have strengthened and clarified the methodology and deliverable of this manuscript. As the editor and all reviewers acknowledged, this study will play a significant catalyst to improve policy/guideline to ensure players’ safety.

**Reviewer #1:**

Abstract

The authors should add the number of days of all practices and games in the 2019 season.

**RESPONSE**: Thank you for the suggestion. We added the number of practices (46), scrimmage (1), junior varsity (9), and varsity games (10) to the abstract, as well as in the study procedure of the method section.

Introduction

[P3, L51] A brief description of the long-term consequences of a sport-related head injury, especially in the context of (high school) football, would be useful.

**RESPONSE**: Excellent point. We have had a long discussion about this point prior to the initial submission whether to mention the potential long-term consequences (e.g., CTE, dementia). Although many data are beginning to point to the adverse long-term outcome, we are also aware of several opposing lines of research. This manuscript does not assess neurologic consequences. Instead, our objective was to examine head impact exposure by levels-of-contact; therefore, we made a conscious decision not to dive into long-term neurologic affects. We instead included some of the acute/subacute (e.g., season-long head impact) neurologic outcomes in the 2^nd^ paragraph of the Introduction. We would like to maintain this stance if that is acceptable. If you think we need to include the potential long-term consequence (e.g., CTE), please let us know.

Methods

[P5, L109] How was an active football team member defined? The authors should describe the inclusion criteria in more detail.

**RESPONSE**: Clarification of this was added to the first paragraph of the methods section.

[P6, L118] What role does the determination of race/ethnicity play in the study and what conclusions can be drawn from the results? If these variables are not informative, they should be excluded.

**RESPONSE**: This is an important point. We listed the lack of generalizability to the general population as the first weakness in our limitations section due in part to the small sample size and lack of racial and diversity (i.e., primarily white males). Because of lack of racial and ethnical diversity, we were unable to conduct any analysis to identify whether race/ethnicity played a role in head impact exposure (i.e., White players tend to sustain more/less head impacts than black and other race). However, we felt as though it is important to state that we were working with a primarily white sample because future research may have different findings/conclusions when working with different race/ethnicity samples. Especially because this is first of its kind to delineate level-of-contact influence on head impact exposure, we think it is important to report race/ethnicity data. We expanded this aspect in the limitation section.

[P6, L122] The observation period from pre-season training camp to the end of the season should be stated with the exact number of days and average minute duration on which the practice was performed.

**RESPONSE**: Thank you for pointing this out. We added the exact number of observed practices and games along with the mean (SD) minute duration to the line you suggested.

[P6, L128] Why were no quarterbacks included in the study? The authors should state reasons for the exclusion.

**RESPONSE**: Excellent point to clarify. There was only one quarterback in the entire high school program during the study enrollment period, and unfortunately, he declined to participate in the study due to his lack of interest and reluctancy to wear the Vector mouthguard. We have clarified this point in the manuscript per your request.

[P8, L178] Why was the total number of head impacts split into groups of 25-60 g, 60-100 g or >100 g? The authors should add a short explanation why this categorization was chosen.

**RESPONSE**: Thank you for this comment. We clarified these exploratory thresholds in the line suggested, by explaining that we modeled Barber-Foss et al. 2019 and Bailes et al. 2013 that suggested < 25 *g* as minimal or negligible magnitude; Guskiewicz et al. 2007 that introduced 60 *g* as a cut-off threshold to induce concussion, whose nuance was softened after many papers reporting that concussion can occur head impact below 60 *g*. Nonetheless, we thought this was a relevant threshold to descriptively present our data. Lastly, Beckwith et al. 2013 reported that average head impact magnitude that led to concussion diagnosis was 100 *g* (precisely, 102.5 + 33.8 *g*). These points are now summarized in the revised manuscript.

Results

[Table 1] The variables “Playing position, n (%)” seem to have the same meaning as the first variable "n". Perhaps the authors could simply put the percentage in brackets after the variable "n" to make the table clearer.

**RESPONSE**: We have implemented this suggestion.

The authors might also consider referring to the following articles:

Crisco JJ, Wilcox BJ, Beckwith JG, et al. Head impact exposure in collegiate football players. J Biomech. 2011;44(15):2673‐2678.

Martini D, Eckner J, Kutcher J, Broglio SP. Subconcussive head impact biomechanics: comparing differing offensive schemes. Med Sci Sports Exerc. 2013;45(4):755‐761.

**RESPONSE**: We added the Crisco article as a reference as it pertains to suggesting significant variance in head impact exposure between position groups. We also included Martini et al paper to suggest the potential interaction between playing style (run-first vs. pass-first offense) and level-of-contact drills. Thanks for the suggestion.

Reviewer #2:

1. For the subsection about ‘Head impact measurement’ (p. 6-7), please add some pertinent information about the reliability and validity of the measurement tool (i.e. Vector mouthguard).

**RESPONSE**: Thank you for pointing this out. We added a comment in the limitations section about the lack of literature validating the Vector mouthguard for true positive versus false positive head impacts, although our unpublished internal validation from our previous study^1,2^ yielded 85-90% agreement between Vector mouthguard and film analysis. Additionally, we added a statement in the Head impact measurement section on the reliability of movement in sensor-installed mouthguards compared to skin patch and skull cap head impact devices.

1.	Zonner S, Ejima K, Bevilacqua ZW, Huibregtse ME, Charleston C, Fulgar CC, Kawata K. Association of increased serum S100B levels with high school football subconcussive head impacts. *Front Neurol.* 2019;10:327.

2.	Zonner SW, Ejima K, Fulgar CC, Charleston C, Huibregtse ME, Bevilacqua ZW, Kawata K. Oculomotor response to cumulative subconcussive head impacts in high school football players: a pilot longitudinal study. *JAMA Ophthalmol.* 2019;137(3):265-270.

2. For Table 1, the row with sex can be omitted. The proportions by position group can be moved to the row with sample sizes. It is unnecessary to report age, BMI, experience, PLA, and PRA using two decimal places. It gives a false sense of precision of the measurements. I think one decimal place will suffice.

**RESPONSE**: These are excellent points. We have made the requested revisions to Table 1.

3. For the subsection on ‘Average head impact magnitudes by levels-of-contact and position group’ (p. 11), the word ‘average’ is ambiguous. I suggest making is clearer that all the values are medians.

**RESPONSE**: Thank you for identifying this ambiguous term, we have changed average to median in this section.

4. For Figure 2, please make it clear that the plots show median number of impacts per player. Presumably the error bars indicate the IQR. In the footnotes, please ensure that all p-values greater than 0.001 are reported with and equal symbol (i.e. not inequality symbol).

**RESPONSE**: Thank you for noticing this. We have made the suggested revisions in the legend and fixed the equal symbols.

5. For Figure 3, please make it clear that the plots show median number of impacts per player. Presumably the error bars indicate the IQR.

**RESPONSE**: Thank you for noticing this. We have made the suggested revisions in the legend.

6. For Figure 3, please make it clear that the plots show median number PLA and PRA. Presumably the error bars indicate the IQR.

**RESPONSE**: Thank you for noticing this. We have made the suggested revisions in the legend.

**Reviewer #3:**

Minor Issues:

1. We would recommend including the research question and hypotheses in the Abstract.

**RESPONSE**: In addition to the objective, we have revised the abstract to include the aspect of our research question aiming to determine which drills had the greatest head impact exposure, subsequently stating our primary hypothesis. Thank you for the suggestion.

2. We would also recommend giving the dates the mentioned rule-changes and recommendations for reducing head trauma frequency in high-school / college football were established (levels-of-contact, tackle football, kickoffs rules) for a better overview on the subject and current stand (lines 56-65 and 74-79).

**RESPONSE**: Thank you for these suggestions. We have added years to two of the initiatives mentioned in the introduction.

3. The authors give the number of previous concussions in Table 1 (line 193), however, they do not describe in the manuscript the relevance of their presence or absence; we would recommend to also meet this topic of risk potentiation in repetitive head impacts.

**RESPONSE**: Indeed, previous concussion history was not included in the analysis as a covariate, since our outcome measure is head impact exposure rather than neurologic outcome. Yet, this is an important point to discuss; therefore, we added a short paragraph elaborating the potential intersection between concussion and subconcussive head impacts right before the clinical implication paragraph in the discussion. Thank you for the suggestion.

4. The authors also give in Table 1 (line 193) in detail the race and ethnicity of the players, although 88% of the players are white and only 4% American Indian and 8% multiracial. Moreover, they do not explain in the manuscript potential interracial differences in concussion risk. We would recommend to either add this information or to shorten the table regarding this aspect.

**RESPONSE**: Excellent point. Reviewer #1 also pointed this out and we expanded the limitation section to acknowledge our lack of racial and ethnical diversity. We decided to keep this race/ethnicity in the table so that future research is encouraged to settle this unsolved question.

Reviewer #4:

Material and Methods

line 123: this is a short season from mid-August 2019 to end of October 2019 for preseason training to the end of season?

**RESPONSE**: This is a great question. It was shorter season than expected because they lost out of the season earlier than expected on Friday, November 1^st^. Had they won a playoff game, the season could have lasted more weeks. There first varsity game of the season was on Friday, August 23^rd^.

Results

line 197: the results are clearly describing the level of contact and head impact exposure. However, what is missing, is how much time the athletes were performing activities of the different contact levels. e.g. if most time was spent doing live contact, it is no wonder that most head impact was generated during this typ of impact. it would be optimal to also include exposure time...

**RESPONSE**: Thanks for pointing this out, we agree. We were sure to include this at the end of our limitations section and made an addition of “time spent in each level of contact.”

A future direction for this line of research is to examine subject specific head impact data that also incorporates time, and more importantly repetitions participated in. Part of the issue with team-wide contact guidelines/restrictions is that there is such large variance between players in both practice and game play participation.

For example, as you can imagine, in any given hour of certain drill one player may experience 50 repetitions of drills and associated head impacts, whereas another player may experience only 0 to 10 repetitions. This creates a significant flaw for team-wide restrictions that are based on contact time rather than repetitions.

Discussion

line 231: also dependent on exposure time, see comment above...

**RESPONSE**: Thank you again, this is an important limitation of the study. See comment above too. Time is an important variable to consider and we would like to see it added as part of future research designs exploring intensity levels. Despite this limitation, and our previous comment above, ultimately the outcome of cumulative head impact exposure per level-of-contact is still an important finding even without the amount of time involved. For example, policy makers and other stakeholders should still be interested in which levels have the most head impact exposure associated with them and could potentially utilize those levels to decrease exposure.

line 233: the skill group received much less cumulative impacts 130 vs over 200. Although no statistically significant finding, don't you think that receiving 30% less impacts does not count? please discuss! otherwise please add power analysis and sample size calculation etc... please see also line 254 where you say so, that skill positions have leas head impact

**RESPONSE**: Thank you for pointing this out. It is, indeed, a noticeable difference in head impact experiences between linemen/hybrid vs. skill. There are some heterogeneity in within-group head impact exposure where some skill players sustain many hits while several linemen experienced less hits, which points to the issue of lack of sample size. We elaborated this issue (clinical significance vs. statistical significance) in both the 1^st^ and 2^nd^ paragraphs of the discussion. This is a great point and we appreciate your suggestion.

line 299ff: another limitation is that exposure time of the different levels of contact is not reported...

**RESPONSE**: We added this revision more clearly in two statements within the limitations section. Thanks for your emphasis of this point.

line 318: usually references are not cited in the conclusion, since the conclusions are your main findings and not the findings of other studies (since you published an original study)

**RESPONSE**: Thanks for identifying this irregularity. We thought about this and it is our hope that we could leave these references in the conclusions as a reminder to the audience that how our results may play a catalytic role to fill the current gap in our knowledge. We would really like to hammer home the point as part of our conclusion that greater dissection of football structure and guidelines may lead to measures that more effectively decrease subconcussive head impact exposure. To balance your perspective, the conclusion section within the abstract does not contain such referential statement. We hope that you will understand our rationale.

Tables

Table 1:please remove psychiatric conditions, since there are none. makes the table smaller and easier to read... also remove the lines "playing position, n (%)", since this information is redundant. the cumulative impact count is actually "average cumulative impact count"? also for the peak linear and rotational acceleration?

**RESPONSE**: We have made these revisions and significantly simplified Table 1.

Reviewer #5: For me as a neurologist it is very important that efforts are made to reduce the cumulative exposure of subconcussional cerebral microtrauma in adolescent high school football players. To verify the validity of the concept of different levels-of-contact which has been establishes by USA Football, the authors present an interesting study that compares the real exposure to mild cranial trauma at different levels-of-contact and different positions of football players. In this respect, the authors prove and validate the intuitive assumption that the different increasing levels-of-contact reflect a hierarchy of cumulative exposure to hits to the head. I have no objections to or suggestions for improvement of the manuscript.

**RESPONSE**: Thank you for reviewing our article, we appreciate your feedback and interest from neurologist standpoint. This is encouraging for us to hear.
