# Supplementary material for: Subconcussive head impact exposure between drill intensities in U.S. high school football
Source: PLoS One. 2020 Aug 14;15(8):e0237800. doi: 10.1371/journal.pone.0237800 (PMC7428124; doi:10.1371/journal.pone.0237800)
Supplement: Attachment — Submitted filename: R2 Response to Reviewers.docx [file pone.0237800.s006.docx]

**Response to Editor/Reviewer 2**

Thank you for your thorough attention to important details. As pointed out, the conclusion section was revised to reflect reviewers’ phrasing as well as removal of references and its sentence all together, as suggested by the editor. Abstract’s conclusion section also reflects these changes.

Additionally, in the first paragraph of result section, we inserted some comparative information about g-force using an example of car crash, which is often used as a referential example in head trauma research. However, there are no good real-life examples about rotational acceleration (krad/s^2^), and this rotational acceleration is almost always correlated to g-force in concussion research (if player experience high, cumulative g-force, then their rotational acceleration (krad/s^2^) is high. Therefore, the car crash example for g-force should suffice to sharpen awareness of the medical personnel.

Thank you.

Keisuke Kawata
